# Possible transport pathway of diazotrophic *Trichodesmium* by Agulhas Leakage from the Indian into the Atlantic Ocean

**DOI:** 10.1038/s41598-024-53297-5

**Published:** 2024-02-05

**Authors:** Bettina Martin, Rolf Koppelmann, André Harmer, Rene-Marcel Plonus

**Affiliations:** Institute of Marine Ecosystem and Fishery Science, Universität Hamburg, Hamburg, Germany

**Keywords:** Biogeochemistry, Ocean sciences

## Abstract

Diazotrophic cyanobacteria such as *Trichodesmium* play a crucial role in the nitrogen budget of the oceans due to their capability to bind atmospheric nitrogen. Little is known about their interoceanic transport pathways and their distribution in upwelling regions. *Trichodesmium* has been detected using a Video Plankton Recorder (VPR) mounted on a remotely operated towed vehicle (TRIAXUS) in the southern and northern Benguela Upwelling System (BUS) in austral autumn, Feb/Mar 2019. The TRIAXUS, equipped with a CTD as well as fluorescence and nitrogen sensors, was towed at a speed of 8 kn on two onshore–offshore transects undulating between 5 and 200 m over distances of 249 km and 372 km, respectively. *Trichodesmium* was not detected near the coast in areas of freshly upwelled waters but was found in higher abundances offshore on both transects, mainly in subsurface water layers down to 80 m depth with elevated salinities. These salinity lenses can be related to northward moving eddies that most probably have been detached from the warm and salty Agulhas Current. Testing for interaction and species-habitat associations of *Trichodesmium* colonies with salinity yielded significant results, indicating that *Trichodesmium* may be transported with Agulhas Rings from the Indian Ocean into the Atlantic Ocean.

## Introduction

Nitrogen is an essential element in any form of life from bacteria to complex metazoans due to its necessity for protein biosynthesis. It is regarded as the limiting nutrient for phytoplankton production in most regions of the open ocean, especially in the surface waters of the tropics and subtropics^1–3^. The estimated loss of available nitrogen by new production and export from surface waters often exceeds the nitrate flux by diapycnal mixing of deeper water into the euphotic zone^3,4^, especially as the substitution of the lost nitrate from deeper layers is a slow process in stratified waters. It is assumed that biological fixation of atmospheric nitrogen (N_2_) by diazotrophic cyanobacteria balances this loss in nutrient-poor regions, fuelling up to half of the new production^5^ and therefore playing an important role in carbon uptake in the marine environment. Shao et al.^6^ compiled a database of estimated global oceanic N_2_ fixation rates.

Cyanobacteria of the genus *Trichodesmium* are considered to be the dominant N_2_ binding organism in tropical and subtropical oceans, affecting the influx of new nitrogen in global marine ecosystems^3,7–10^ by making up for 30–80% of oceanic N_2_-fixation rates^11^. Trichomes of *Trichodesmium* can occur as macroscopic aggregates and can produce enormous blooms; for example, the red tides caused by *Trichodesmium erythraeum* give the Red Sea its name. As the N_2_ binding enzyme nitrogenase is rapidly inactivated by O_2_, other diazotroph bacteria separate N_2_ fixation from O_2_ evolving photosynthesis either in time, i.e., N_2_ fixation by night, or in space by forming heterocysts to protect the enzyme. However, *Trichodesmium* is a nonheterocystous bacterium that performs N_2_ fixation in the daytime. Bergmann and Carpenter^12^ found spatial nitrogenase sequestration mechanisms in *Trichodesmium*, a cell type subsequently termed diazocytes.

While most of the studies describe N_2_ fixation in tropical and subtropical regions of the open ocean^2,3,5,13–16^, only little is known about the contribution of diazotrophic bacteria to the nitrogen budget in coastal waters^17–19^ and upwelling systems^20–25^. A recent study by Tang et al.^19^ showed unexpectedly increasing underway N_2_-fixation rates from the oligotrophic Sargasso Sea to American coastal waters with the highest activities close to the coast, and some of the N_2_-fixation hotspots coincided with high phosphorus concentrations near the surface.

**Figure 1:**
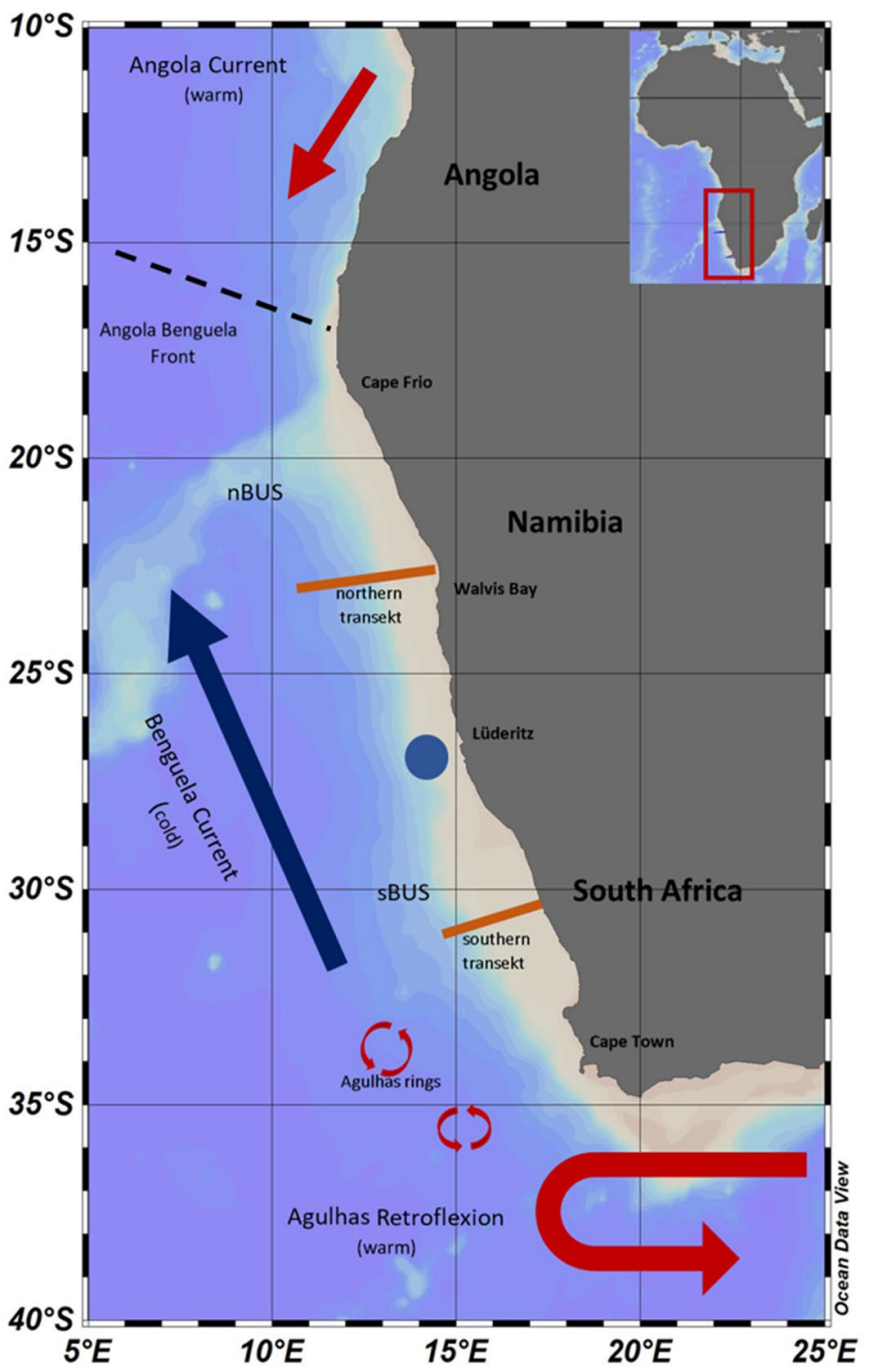
The Benguela Upwelling System. The blue circle marks the Lüderitz upwelling cell, dividing the northern and southern BUS. The map was created with the Ocean Data View software, version 5.6.3, https://odv.awi.de/.

We investigated the distribution of *Trichodesmium* in the Benguela upwelling system (BUS), one of the most productive upwelling systems in the world^26^. It is located off the west coast of southern Africa, bordering Angola, Namibia and South Africa (Fig. 1). The productivity of the system is driven by strong alongshore trade winds and upwelling of cold, nutrient-rich water into the euphotic zone. It is traditionally divided into a northern and a southern subsystem^27–29^. The northern BUS (nBUS) is located between the Angola-Benguela Front (14° S to 16° S), and the Lüderitz upwelling cell at approximately 26.30°S^30^ is influenced by South Atlantic Central Water (SACW), intruding from the Angola Gyre. The southern BUS (sBUS), fed by Eastern South Atlantic Central Water (ESACW) flowing in from the Cape Basin, is limited in the south by the warm Agulhas Current at 34°S. The Agulhas Current is a strong warm and salty western boundary current originating in the southwestern Indian Ocean, following the continental shelf from Maputo to the Agulhas Bank. When the current leaves the shelf and turns eastward into the Agulhas Retroflection, rings detach and move into the South Atlantic Ocean^31–33^. This ‘Agulhas Leakage’ strongly varies on interannual to decadal timescales^34^. Marshall et al.^35^ traced water mass circulations in the Agulhas Current using nitrate isotopes. They stated that nitrogen fixation occurs in the Agulhas Current and rather low δ^15^N signatures indicate Agulhas Leakage into the South Atlantic. However, little is known about the interoceanic transport pathways of plankton organisms such as *Trichodesmium* in these rings or eddies. *Trichodesmium* colonies were already found in high numbers in the southwest Indian Ocean, south and east of Madagascar in February 2005^36^ and even in the Agulhas Current region^37^.

Reports of the presence of *Trichodesmium* or indications of N_2_ fixation in the BUS are inconsistent. While Wasmund et al.^21^ found very low abundances of *Trichodesmium* and no evidence of N_2_ fixation in the nBUS, Sohm et al.^20^ reported elevated N_2_ fixation rates in or near the Benguela Current compared to the oligotrophic South Atlantic Gyre. The authors found that high NO_3_^-^ concentrations did not exclude N_2_ fixation in upwelling areas.

A remotely operated towed vehicle (TRIAXUS), equipped with several sensors and an attached Video Plankton Recorder (Seascan VPR)^38^, enables simultaneous measurements of biogeochemical and physical water mass properties combined with the occurrence of planktonic organisms over large distances. Because *Trichodesmium* colonies are easily damaged by conventional net hauls, Niskin bottles or buckets^39^, noninvasive techniques such as underwater camera systems seem to be more appropriate to study these objects. By using the TRIAXUS mounted VPR, we will provide the first evidence of possible interoceanic transport pathways of *Trichodesmium* from the Indian Ocean into the Atlantic Ocean as well as provide information about its distribution in the Benguela upwelling region.

## Results

**Figure 2:**
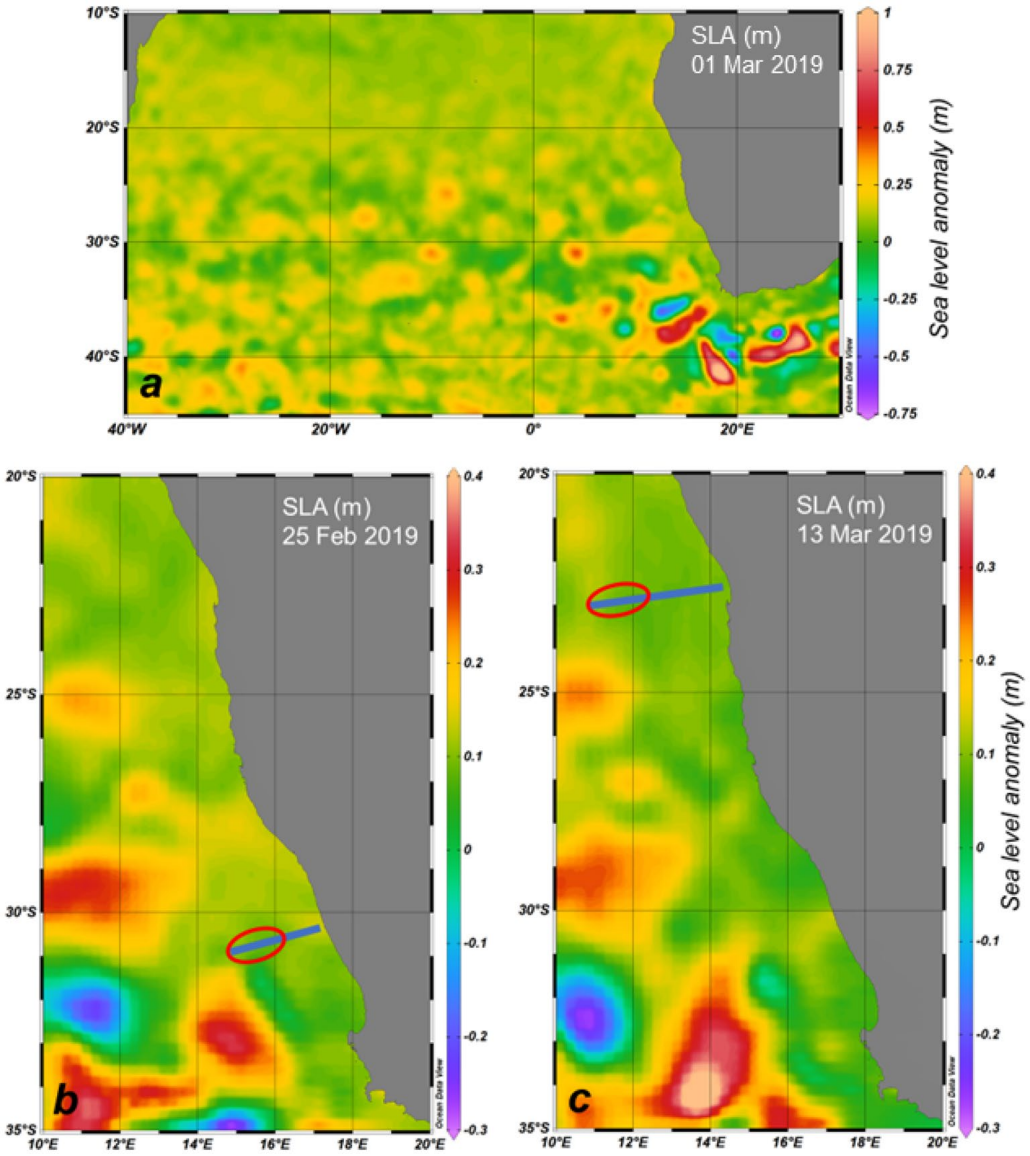
Overview of sea level anomalies (SLA) on the 1st of March 2019 (**a**) from satellite data and with studied transects (blue line) and occurrence of *Trichodesmium* colonies (red frames) on the 25th of February 2019 in the sBUS (**b**) and 13th of March 2019 in the nBUS (**c**) during sampling.

The satellite observations of sea level anomalies (SLA) around Southern Africa demonstrate that eddy structures originating from the Agulhas current propagated to the north and north-west during the sampling time (1st of March 2019, Fig. 2a). The SLA and the occurrence of *Trichodesmium* colonies at the dates of sampling showed enhanced abundances of colonies adjacent to dynamic structures associated to eddies (Fig. 2b, c).

**Figure 3:**
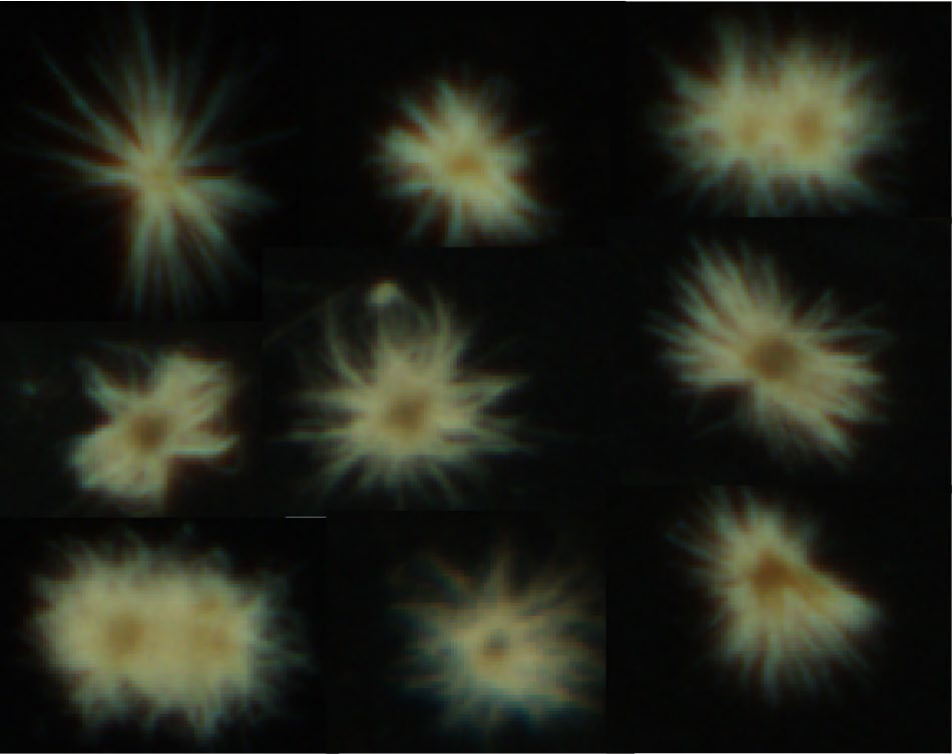
VPR frames containing pictures of one or two *Trichodesmium* colonies as detected by trained software.

**Figure 4:**
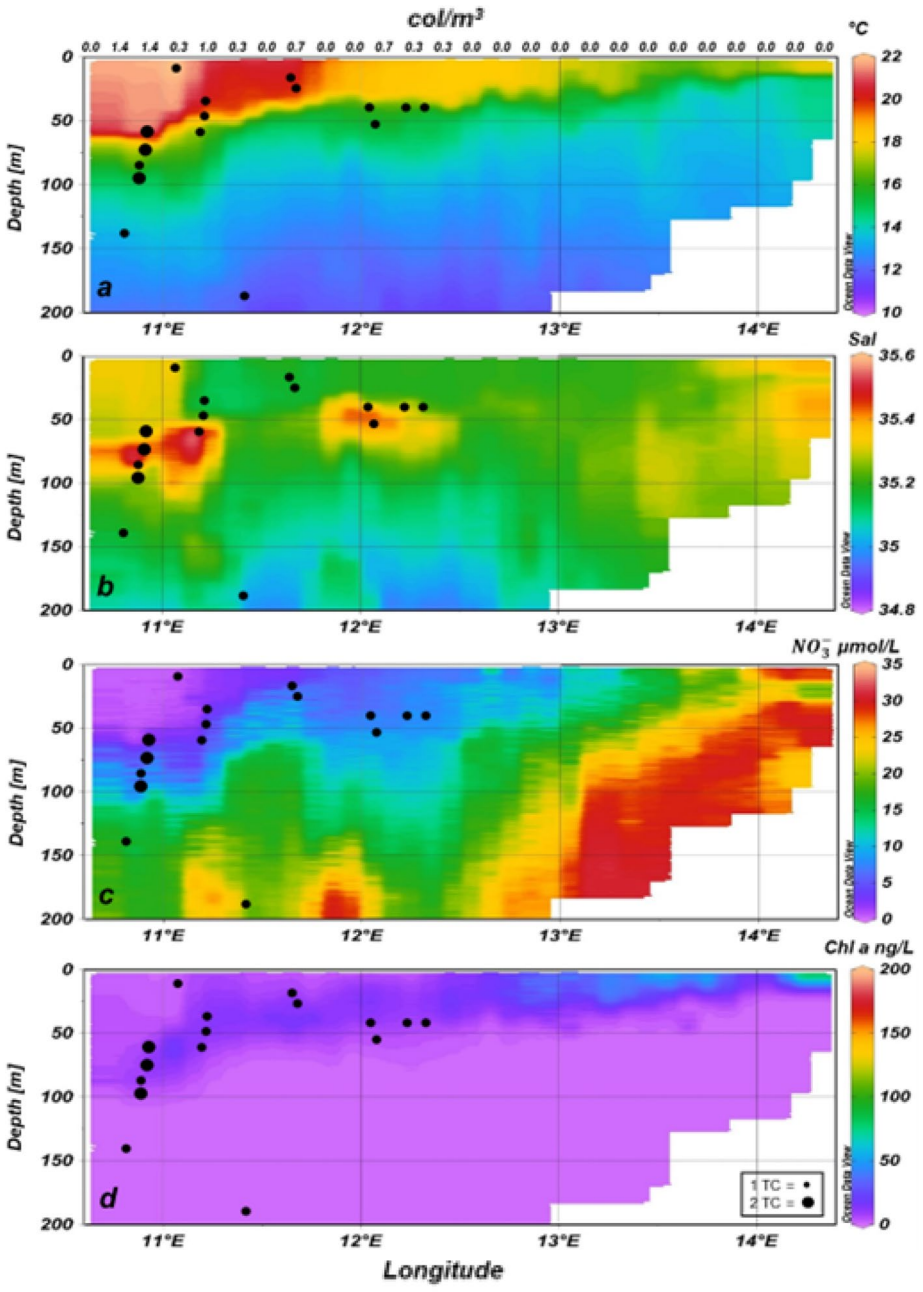
Temperature (**a**), salinity (**b**), nitrate (**c**) and Chl *a* (**d**) data measured by TRIAXUS mounted sensors and occurrence of *Trichodesmium* colonies (black dots) detected by TRIAXUS mounted VPR at an onshore-offshore transect in the nBUS. The number of colonies per black dot is shown in panel *d* at the bottom right. The upper x-axis represents the hourly mean abundance of *Trichodesmium* colonies (col/m^3^).

**Figure 5:**
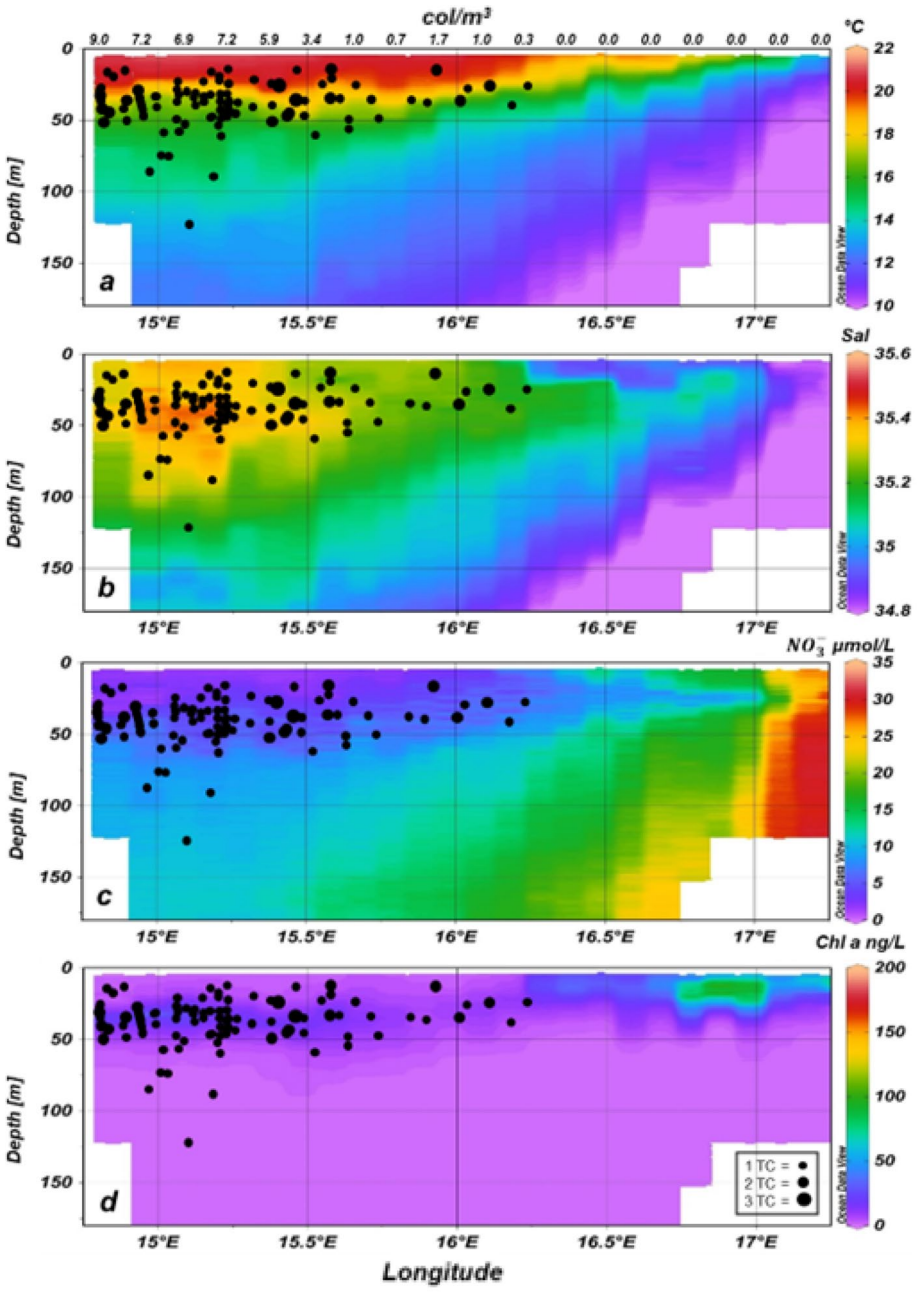
Temperature (**a**), salinity (**b**), nitrate (**c**) and Chl *a* (**d**) data measured by TRIAXUS mounted sensors and occurrence of *Trichodesmium* (black dots) detected by TRIAXUS mounted VPR at an onshore-offshore transect in the sBUS. The number of colonies per black dot is shown in panel *d* at the bottom right. The upper x-axis represents the hourly mean abundance of *Trichodesmium* colonies (col/m^3^).

*Trichodesmium* colonies (Fig. 3) were detected using a VPR in both investigated regions of the BUS, with lower abundances in the nBUS than in the sBUS (Figs. 4a–d and 5a–d).

**Figure 6:**
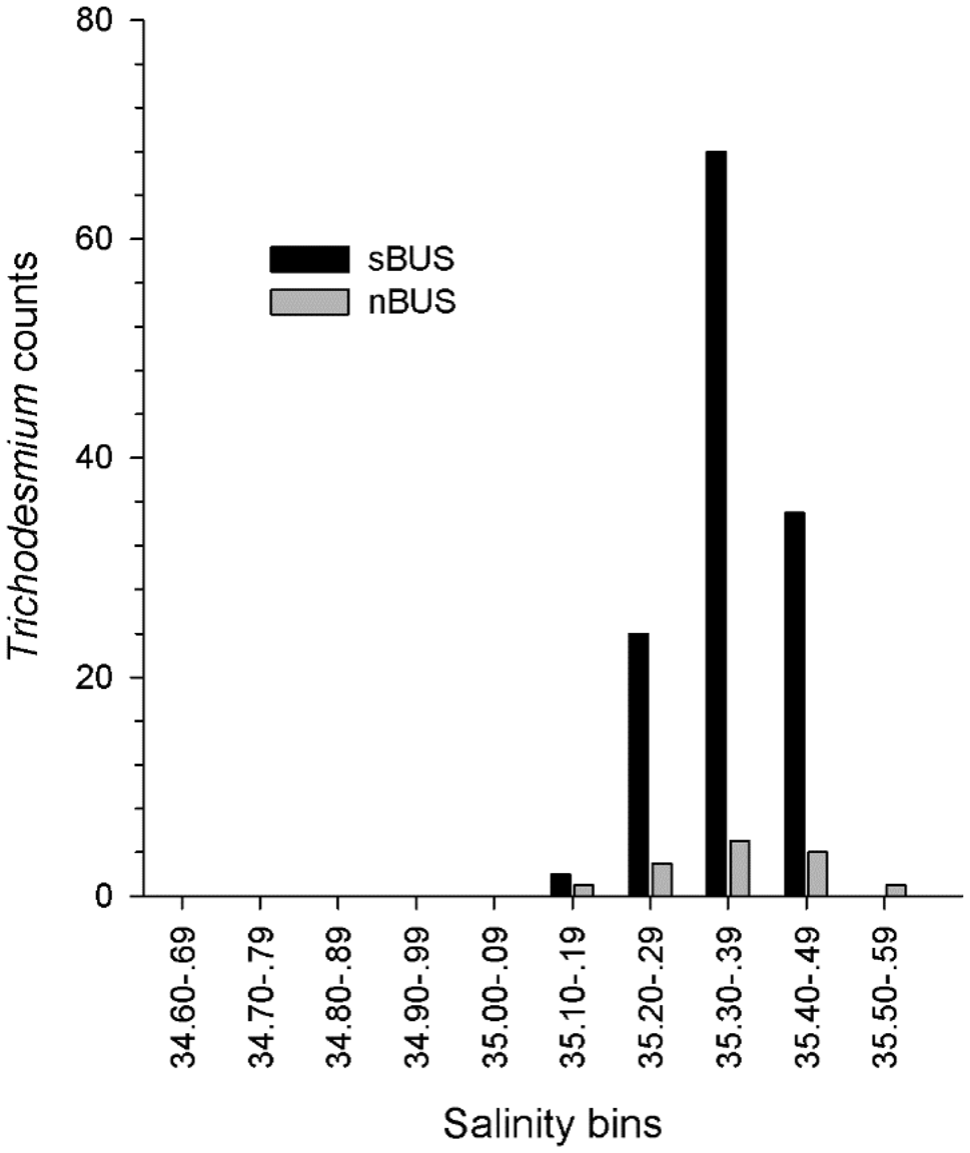
Counts of *Trichodesmium* colonies in the nBUS and sBUS related to salinity bins.

The blue algae occurred rarely at the surface but mostly between 30 and 60 m and occasionally down to 187 m in the north and 137 m in the south, respectively, and in waters of higher salinity > 35.1 (Figs. 4b, 5b and 6). *Trichodesmium* was not detected in nitrate-rich upwelled waters close to the coast (Figs. 4a, c and 5a, c). In the north as well as in the south, chlorophyll *a* (Chl *a*)values were slightly higher in the vicinity of the colonies (Figs. 4d, 5d).

A generalized additive model (GAM) indicated that salinity (p < 2 × 10^–16^) and temperature (p = 1.32 × 10^–6^) are significantly related with *Trichodesmium* colonies (see Appendix Table Suppl. 1). The model explained 35.5% of the observed deviance in the *Trichodesmium* distribution. Since the temperature signal of Agulhas Rings is disappearing quickly, we decided to use only the more conservative salinity parameter to relate the *Trichodesmium* counts with environmental variables (Fig. 6).

## Discussion

A TRIAXUS mounted VPR proved to be a suitable gear to detect a fragile organism like *Trichodesmium* within its natural habitat: colonies could be quantified without being destroyed, and environmental data could be measured simultaneously, thus making it possible to relate these data to the occurrence of the organisms. Other approaches, as observed in studies by Sandel et al.^40^ and Dupouy et al.^41^, involved the utilization of an Underwater Video Profiler (UVP) mounted on a CTD. While this method also provides environmental data during the detection of *Trichodesmium* colonies throughout the water column, it is constrained by its stationary sampling strategy when compared to the continuous data acquisition capability of the towed TRIAXUS-mounted system. Previous investigations by Dupouy et al.^41^ reported peak concentrations up to 7093 col/m^3^ in the oligotrophic Southwestern Pacific. Sandel et al.^40^ measured 9.4 × 10^4^ col/m^2^ at 23° W, 5° N, while Fernandez-Carrera et al.^16^ found 3000 col/m^3^ below the surface in the Guinea Dome region (9° N–15° N) using an UVP. Such high abundances of *Trichodesmium* are likely to occur in tropical oligotrophic oceans. In our study, from a more productive region, we observed lower concentrations ranging from 3 to 9 col/m^3^. Notably, these findings align with those of Davis and McGillicuddy^42^, who used a VPR and reported around 35 col/m^3^ in the western Atlantic Ocean and 6–7 col/m^3^ in the eastern Atlantic Ocean.

Recently the development of algorithms for the detection of *Trichodesmium* mats by satellite^43^ made it possible to screen much larger areas to quantify the organisms than it would have been possible by sampling from onboard ships, but this method can only provide information about blooms in surface waters.

The low surface temperatures and increased nitrogen values near the coast compared to offshore waters indicate that coastal upwelling had taken place recently in both regions of the BUS, with a stronger signal in the sBUS. It was apparent that *Trichodesmium* was absent in these cold, nutrient-rich onshore waters. Colonies were found further offshore in subsurface and deeper water layers at temperatures below 18 °C on both transects but with distinctly higher abundances in the sBUS. Our data showed, in contrast to other investigations^14,44,45^, that *Trichodesmium* was not limited to areas of warm water > 20 °C. However, the abundance frequency was highest in water lenses with comparatively high salinity (see also Fig. 6).

The nBUS and sBUS are fed by different source waters, causing lower salinities of < 35 in the south due to the inflow of ESACW from the Cape Basin. The fact that the water lenses showed much higher salinities makes it likely that the salt anomalies are caused by Agulhas Rings, which transport their persisting salt content to the South Atlantic^46^, even though they lose their anomalous surface thermal content quickly to the atmosphere^47^. Agulhas Leakage was detected by sea surface anomalies in the sBUS (Fig. 2) and tracks of Agulhas eddies and leakages into the investigated area and further into the western South Atlantic are published by Raith et al.^48^, Guerra et al.^49^ and Wei and Wang^50^. Water with an Agulhas signature was even found at the western side of the Atlantic^49^. We assume that these eddies taking the *Trichodesmium* colonies northwards to the offshore regions of the Benguela Current. Such a mechanism of colony transport has been previously reported. Davis and McGillycuddy^42^, described increased colony concentrations in warm and salty water of anticyclonic eddies during an east‒west transect in the North Atlantic but could not elucidate the underlying mechanisms of these findings. Sandel et al.^40^ also investigated the *Trichodesmium* occurrence in the Atlantic Ocean, using an UVP 5 on a transect at 18°N and 20°-27°W, and found a *Trichodesmium* peak of 5.5 × 10^4^ col/m^2^ in an anticyclonic eddy (20°W). Groninger et al.^51^, who studied a Gulf Stream frontal eddy, which originated from waters of higher productivity, found differences among the microbiom in and outside of the eddy with lower counts of *Trichodesmium* in the eddy, compared to Gulf Stream waters. The eddy-associated microbial community occupied a larger area than identified by temperature and salinity alone. If we assume such transport by Agulhas Rings, the microbiome of the Leakage may also have occupied a larger area and lasted longer than suggested by the environmental signals.

Similar to our study, Sandel et al.^40^ as well as Davis and McGillycuddy^42^ detected higher densities of *Trichodesmium* colonies in subsurface depths. Sandel et al.^40^ stated that the *Trichodesmium* bloom extended even down to 80 m with a peak at 40 m depth. Davis and McGillicuddy^42^ assumed that the high abundances they found in deeper water indicated that previous calculations underestimated the N_2_-fixation rates of *Trichodesmium,* and a correction of these calculations might even explain the missing nitrogen in the global nitrogen cycle. This would make *Trichodesmium* a crucial organism for primary production in the world oceans. However, increased nitrate values in the vicinity of the colonies were not measured in our study, whereas slightly higher Chl *a* concentrations in the vicinity of *Trichodesmium* accumulations were detected. Whether this increase in chlorophyll is caused by or partly by *Trichodesmium* can only be speculated. Another reason for the sinking of the Chl *a* maximum with increasing distance from the coast, especially in upwelling regions, could be a deeper Chl *a* maximum because the penetration depth of light increases with decreasing suspended matter offshore.

In the research conducted by Wasmund et al.^21^, spanning seven cruises and 66 stations in the northern Benguela Upwelling System (nBUS), *Trichodesmium* was observed at only two stations. The sporadic presence of *Trichodesmium* could potentially be attributed to the dynamic interplay of eddies transporting organisms from the Indian Ocean to the BUS. This patchy distribution may find its explanation in the significant interannual to decadal fluctuations in Agulhas Leakage^34^. Notably, Biastoch et al.^33^ documented a rising trend in Agulhas Leakage, driven by the strengthening of Southern Hemisphere westerlies. Model simulations further anticipate a sustained increase in Agulhas Leakage, propelled by the intensification of westerlies due to global warming^52^. This, in turn, suggests the possibility of an increased *Trichodesmium* abundance in the BUS, originating from colonies in the Indian Ocean.

## Conclusion

A Video Plankton Recorder mounted on a towed and undulating TRIAXUS proved to be a suitable gear to study the fragile colonies of the diazotroph cyanobacterium *Trichodesmium*. *Trichodesmium* was detected in low numbers in the nBus and in higher numbers in the sBUS in austral summer. However, colonies were absent in recently upwelled surface water, indicating a different source water mass for these cells. The higher abundance in water lenses of elevated salinity compared to surrounding waters provides evidence that detached Agulhas Rings moving northward may transport *Trichodesmium* from the Indian into the Atlantic Ocean. We provide evidence of this pathway by detecting N_2_-fixing *Trichodesmium* in the Benguela Current region associated with water masses with an Agulhas signature. However, it cannot ruled out that *Trichodesmium* colonies were of different origin and their association to water of relative high salinity was caused by an undetected trapping effect.

## Material and methods

During a cruise with the research vessel METEOR (M153) in February and March 2019^53^, two onshore-offshore transects in the nBUS and sBUS were examined (Fig. 1, Table 1). The expedition took place as part of the TRAFFIC project (Trophic transfer efficiency in the Benguela Current^29^). In addition to other devices, a TRIAXUS equipped with a CTD, a video plankton recorder (VPR) and a nitrate sensor was deployed (see below). Sea level data were obtained from Global Ocean Gridded L4 Sea Surface Heights and derived variables were reprocessed (COPERNICUS CLIMATE SERVICE) ﻿10.48670/moi-00145.

**Table 1:** Specifications of studied transects.

Date	Station	Haul	Region	Time UTC	Lat	Long	Distance (km)	No of oblique profiles
25 to 26 Feb 2019	19-3	4 + 5	sBUS	15:25 to 09:18	−31.00 to −30.39	14.82 to 17.26	249	194
12 to 13 Mar 2019	56-2	9	nBUS	20:13 to 21:33	−22.60 to −23.00	14.30 to 10.76	372	270

### Remotely operated towed vehicle—TRIAXUS

**Figure 7:**
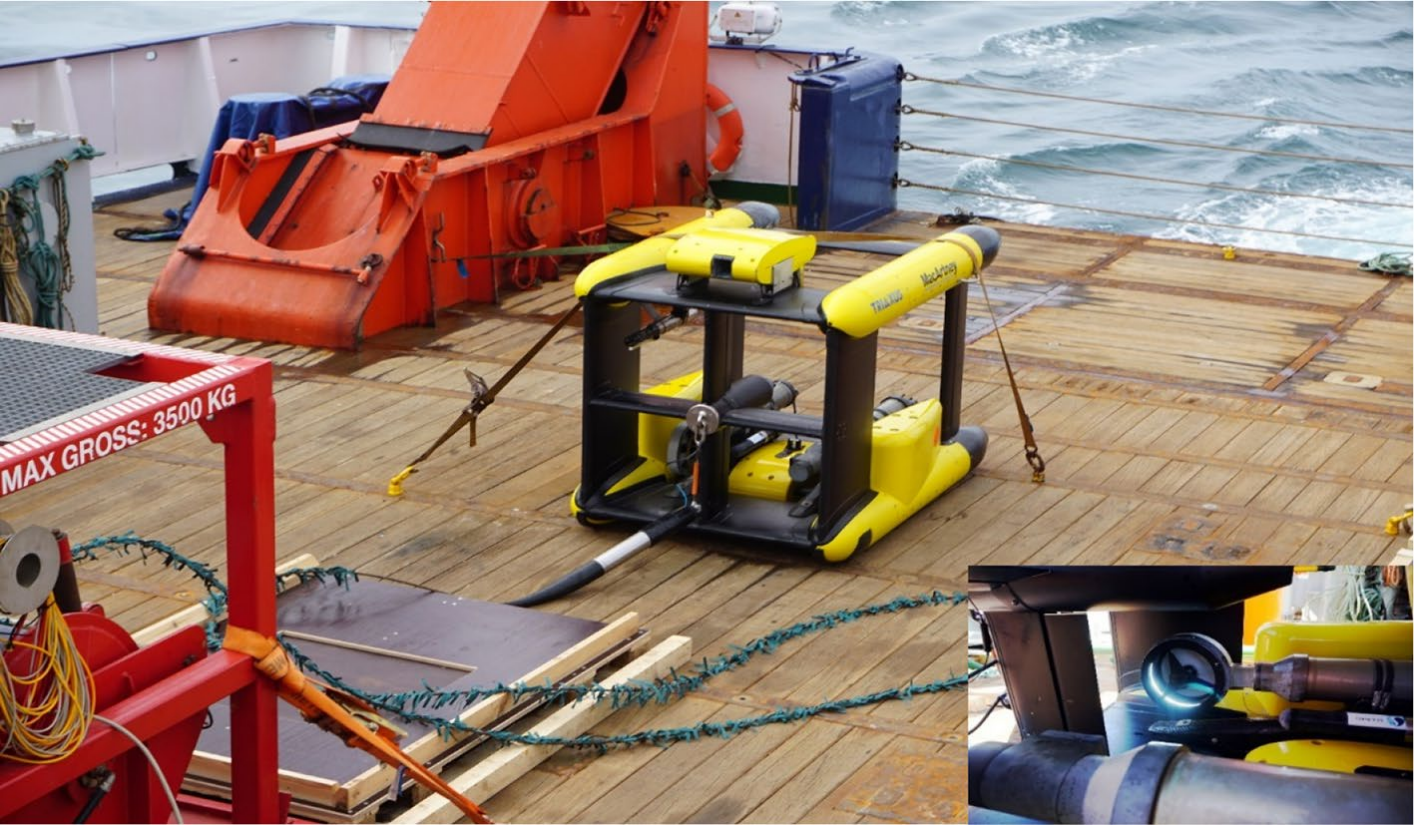
TRIAXUS system on board of RV Meteor with a detailed view of the Video Plankton Recorder with flashing strobe (bottom right).

The TRIAXUS ROTV (Remotely Operated Towed Vehicle, MacArtney, Fig. 7) was used to conduct high-speed large-scale measurements. The TRIAXUS is a towfish with a possible towing speed between 2 and 10 knots and a vertical speed of up to 1 m to undulate between 1 and a maximum of 350 m depth. A lateral offset of up to 80 m to either side of the ship makes it possible to avoid disturbance from the wake of the ship during measurements^54^. The ROTV was towed at a speed of 8 knots (4.1 m s^-1^), undulating with a vertical speed of 0.1 m s^−1^ from 5 m below the sea surface to a maximum depth of 200 m or at least 5 m above the sea floor. In addition to the VPR, the TRIAXUS was equipped with several other sensors, including a pumped Sea-Bird SBE 49 FastCAT CTD, an Aanderaa oxygen optode 4330 F, a Sea-Bird Deep Suna Ocean Nitrate Sensor and a Turner C6 Cyclops sensor for Chl *a*, Phycocyanin, Phycoerythrin and Turbidity (see 10.1594/PANGAEA.964889).

### Video plankton recorder—VPR

The VPR (Seascan) developed by Davis et al.^38^ is a digital underwater camera system equipped with a high-resolution digital camera (Pulnix TM-1040) that records 25 image frames s^−1^. A strobe (Seascan—20 W Hamamatsu xenon bulb) provided the illumination for the images with a pulse duration of 1 μs that was synchronized with the camera shutter. The chosen field of view was 24 × 24 mm with a focal depth of ~ 60 mm at 246 mm from the lens. The image volume was 34.93 ml and the spacing between images was 0.163 m. The northern transect covered a distance of 372 km, and the southern transect covered 249 km, with an hourly mean sampling volume of 2.9 m^3^ h^−1^. In total, a volume of 46.9 m^3^ in the sBUS and 73.5 m^3^ in the nBUS was analyzed. In the sBUS ca. 57,400 particles and in the nBUS ca. 105,700 particles were counted.

Analysis and classification of images and sensor data were sent in real time to an onboard unit via a fiber optic cable. Imaged particles were extracted as regions of interest (ROIs) by Autodeck image analysis software (Seascan Inc.) and saved to the computer hard drive as TIFF files. Each ROI was tagged using a time stamp to allow merging with the hydrographic parameters that were written to a separate logfile.

### Detection of *Trichodesmium* (software)

For automated plankton classification, training and application of deep learning models were performed with a GPU-supported TensorFlow^55^ and Keras^56^ under Python 3.7^57^. Training sets developed by Plonus et al.^58^ using VPR images taken during cruises in the North Sea and Baltic Sea were used and evaluated manually. On the studied images, *Trichodesmium* colonies could be easily recognized due to their unique form and the fact that they were very rarely photographed overlapping with other species. Free trichomes could not be securely identified, which is why only colonies were counted. *Trichodesmium* densities were calculated by taking the calibrated image volume of a single frame multiplied by the number of images taken.

### Statistical analyses

We used a GAM (generalized additive model)^59^ to test for significant interactions between *Trichodesmium* colonies and temperature, salinity, oxygen, Chl *a*, and nitrate. Due to the relatively low number of *Trichodesmium* colonies (sBUS = 129, nBUS = 19) we aggregated our data in 1 m depth layers and 10 km distance sections and tested for presence/absence in the sBUS area only.

### Supplementary Information

Supplementary Table 1.

## Data Availability

All data needed to evaluate the conclusions in the paper are available at 10.1594/PANGAEA.964889.
