# Supplementary material for: Possible transport pathway of diazotrophic Trichodesmium by Agulhas Leakage from the Indian into the Atlantic Ocean
Source: Sci Rep. 2024 Feb 5;14:2906. doi: 10.1038/s41598-024-53297-5 (PMC10844604; doi:10.1038/s41598-024-53297-5)
Supplement: Supplementary file 1 — Supplementary Table 1. [file 41598_2024_53297_MOESM1_ESM.docx]

**Tab. Suppl. 1** Summary of the model output for a binomial gam to test the relationship of Trichodesmium colonies with temperature and salinity. Significance codes: 0 ‘***’, 0.001 ‘**’, 0.01 ‘*’, 0.05 ‘.’, 0.1 ‘ ’.

| Family: | binomial |  |  |  |
| --- | --- | --- | --- | --- |
| Link function: | logit |  |  |  |
| Formula: | trichos > 0 ~ s(temp, k = 3) + s(sal) | | |  |
| Parametric coefficients: |  |  |  |  |
|  | Estimate | Std. Error | z value | Pr(>\|z\|) |
| (Intercept) | -10.32 | 0.9991 | -10.33 | <2e-16 *** |
| Approximate significance of smooth terms: | | |  |  |
|  | edf | Ref.df | Chi.sq | p-value |
| s(temp) | 1.968 | 1.999 | 31.12 | **1.32e-06** *** |
| s(sal) | 1.000 | 1.000 | 38.34 | **< 2e-16** *** |
| --- |  |  |  |  |
| R-sq.(adj) = 0.16 |  | Deviance explained = **35.50%** | |  |
| UBRE = -0.8521 |  | Scale est. = 1 |  | n = 3968 |
